# A traceable authentication system based on blockchain for decentralized physical infrastructure networks

**DOI:** 10.1038/s41598-025-01114-y

**Published:** 2025-05-14

**Authors:** Liang Liu, Kazumasa Omote

**Affiliations:** University of Tsukuba, Tsukuba, Japan

**Keywords:** Computer science, Information technology

## Abstract

With the rapid development of decentralized physical infrastructure networks (DePIN), resource sharing among devices has become more convenient, but it also introduces new risks of malicious behavior. Attackers may evade accountability by frequently switching accounts, making authentication and traceability essential. However, existing approaches face limitations in scalability, cost, and privacy, rendering them unsuitable for large-scale DePIN environments. To address these challenges, this paper proposes a traceable authentication system that integrates government public key infrastructure with blockchain technology. The system leverages government-issued electronic identity cards to generate digital signatures and employs smart contracts to automate the authentication process. Physical infrastructure (PI) devices can directly retrieve authentication results from smart contracts, enabling efficient and secure authentication. Moreover, the immutable logs recorded on the blockchain provide reliable evidence for tracing a user’s unique real-world identity. Performance evaluation results demonstrate that the system reduces authentication costs by up to 40% and achieves high throughput under both RSA and ECDSA algorithms. The contributions lie in enhancing the scalability of DePIN authentication, preserving user privacy, and enabling real-world traceability.

## Introduction

With the continuous advancement of blockchain technology and the widespread deployment of Internet of Things (IoT) devices, Decentralized Physical Infrastructure Networks have begun to attract increasing attention. DePIN refers to an emerging infrastructure paradigm that leverages blockchain to construct and manage physical infrastructure networks, including IoT devices^1^. The decentralized nature of blockchain enables these networks to operate without a central authority, allowing users to share physical and digital resources within a zero-trust environment, such as wireless connectivity, energy, computing power, and bandwidth^2,3^. While blockchain provides DePIN with an efficient management mechanism, its zero-trust architecture also introduces new security vulnerabilities. An attacker in a blockchain network can freely attempt to gain unauthorized access to devices or even damage them. He may also try to exploit the devices to compromise sensitive data, or damage the stability of networ^4,5^. As more IoT devices are incorporated into DePIN, the potential impact of such threats becomes increasingly significant. Therefore, developing robust prevention and governance mechanisms is becoming critical to ensure the scalability and reliability of DePIN across various domains of physical infrastructure.

To address malicious behaviors in DePIN systems, traditional approaches face significant challenges. Particularly when attackers evade accountability by frequently switching accounts, existing detection methods often struggle to effectively govern such malicious behavior^6^. Therefore, DePIN systems must achieve two essential capabilities simultaneously: authentication before use to prevent unauthorized access and identity tracking after use to ensure the accountability of authorized users. On the one hand, existing authentication schemes are not well-suited for large-scale deployment in DePIN environments. First, many resource-constrained devices cannot execute complex verification tasks, necessitating verification processes being conducted on the blockchain^7–9^. Second, this blockchain-based approach requires storing detailed information for each device, creating a dilemma: on-chain storage incurs substantial overhead, while off-chain storage relies on centralized database availability, both limiting the scalability of blockchain-based authentication.

On the other hand, current traceability methods fall short of meeting the demands for both privacy protection and dynamic management. Recording user identities on-chain may enable traceability but introduces risks such as privacy leakage^10^. Anonymous identity schemes, while preserving user privacy, lack the capacity for effective accountability^11^. GPKI offers a balanced approach to traceability and privacy, but its reliance on fixed verification locations and manual procedures limits its flexibility^12^. These technical limitations render existing schemes difficult to effectively apply in the dynamic and evolving DePIN environment. Therefore, there is an urgent need for a traceable authentication framework that can securely, efficiently, and flexibly govern malicious behaviors in DePIN systems.

To address these challenges, government-issued eID cards provide a reliable solution^13,14^. Typically certified by governmental authorities, eID cards utilize cryptographic techniques to provide strong identity assurance. They often incorporate hardware modules capable of generating digital signatures, while accompanying certificates ensure the authenticity of the user’s identity. This approach eliminates the need to store sensitive identity data on-chain, significantly reducing blockchain storage overhead. On blockchain platforms that support smart contracts, identity verification can be performed automatically using public keys and digital signatures^15^. This enables flexible authentication without manual intervention. Moreover, the immutability of verification records on the blockchain, when bound to unique digital signatures, offers reliable evidence for tracing user identities and enforcing accountability in cases of malicious behavior.

In order to address the challenges of preventing and governing malicious behavior in DePIN, this paper proposed a traceable authentication system in which eID cards serve as a bridge between blockchain and GPKI. The system integrates the identity assurance provided by GPKI with the decentralization and automation capabilities of blockchain, thereby mitigating the limitations inherent in each. The proposed approach facilitates automated identity authentication by leveraging smart contracts to verify digital signatures and certificates. Simultaneously, it establishes a verifiable linkage between users’ public keys and real-world identities, enabling traceability without compromising user privacy.

The main contributions of this paper are as follows.

- This paper proposes an authentication system that integrates eID cards with blockchain technology, enabling traceability of users’ unique identity.
- The system avoids storing user identity data on the blockchain, reducing storage overhead while protecting user privacy.
- This paper presents a security analysis demonstrating that the proposed system satisfies identity verifiability, privacy protection, and non-repudiation. The system is robust against identity forgery, replay attacks, and privacy leakage.
- To evaluate system performance, this paper conducts hardware signature efficiency tests using Japanese-issued eID cards and evaluates blockchain processing capacity and gas costs based on both RSA and ECDSA signature algorithms in local and test networks. Experimental results show that the system offers good algorithm compatibility and scalability, reducing authentication costs by 40% compared to existing methods in high-concurrency scenarios.

The structure of this paper is as follows, and Chapter “Related work” introduces the related work. Chapter “Preliminaries” introduces the preliminary knowledge for this system. Chapter “System model and security considerations” introduces the system model and security requirements. Chapter “Proposed architecture” introduces the structure of the proposed system and the steps of authentication. Chapter ““Security Analysis” conducted a security analysis of the system. Chapter “Performance evaluation” evaluates the system performance. Chapter “Discussion” describes the features of the proposed system by comparing it with related work.

## Related work

This research primarily focuses on authentication schemes for resource-constrained devices and authentication mechanisms in GPKI systems. Resource-constrained devices require lightweight and efficient identity authentication solutions, while GPKI emphasizes robust security, identity traceability, and automation. However, both fields face unresolved challenges in simultaneously balancing security, efficiency, scalability, and privacy protection.

### Authentication schemes for resource-constrained devices

Researchers have proposed various methods for secure, lightweight, and efficient authentication. Chen et al.^16^ proposed a lightweight authentication protocol based on blockchain, significantly reducing computation and communication overhead by utilizing simple cryptographic primitives and blockchain technology. However, this approach cannot track the identity of authorized malicious users. Khan et al.^17^ presented a secure data processing architecture called BDLT IoMT. It integrates blockchain and support vector machine models. Although this system achieves secure, traceable, and optimized data sharing among distributed nodes, its limitations restrict its applicability in dynamic security scenarios. Mao et al.^10^ utilized Trusted Execution Environments to ensure data and message security, leveraging smart contracts for lightweight distributed authentication. However, the proposed method stores identity information directly on the blockchain, leading to significant storage overhead. Garba et al.^18^ introduced a lightweight certificate-based authentication scheme by simplifying X.509 certificates and employing blockchain for storage and verification. While this approach reduces data size, it struggles with scalability in large-scale IoT deployments due to persistent storage pressure. Dwivedi et al.^19^ proposed a decentralized security architecture using blockchain smart contracts and the InterPlanetary File System (IPFS). It reduced storage costs but introduced dependencies on network topology and node availability.

Blockchain-based authentication has achieved compatibility and automation in supporting resource-constrained devices. However, storing identity information on-chain incurs substantial overhead that scales linearly with user growth, thereby limiting system scalability. In contrast, off-chain storage in centralized databases introduces dependencies on third-party infrastructure, undermining blockchain’s decentralization feature and introducing single points of failure. Moreover, robust privacy-preserving mechanisms effectively conceal user identities but hinder the traceability of malicious actions. Conversely, systems prioritizing traceability expose excessive identity-related information, thereby introducing privacy risks.

### Authentication mechanisms in government PKI systems

In GPKI systems, researchers have explored blockchain-based solutions to improve the efficiency of authentication. Khan et al.^20^ proposed an architecture based on blockchain and enhanced intelligent IoT. Although the system has introduced smart contracts and distributed mechanisms in transaction processing and device registration, it still relies on manual verification by system experts and has limited automation. Rahat et al.^12^ proposed an identity management system where keys are stored in smart contracts and identity information resides in an off-chain local database. However, the proposed approach relies on manual verification of database records, introducing inefficiencies and delays. Tang et al.^21^ developed a blockchain-based social security system using smart contracts for authentication and IPFS for identity storage. However, the final verification still requires manual intervention, limiting automation. Paez et al.^22^ combined blockchain technology with biometric identification for electronic identity authentication. While effective for secure e-government services, this system is constrained to deployment within government facilities, reducing its flexibility and accessibility. Liu et al.^15^ proposed an efficient authentication system using smart contracts. However, the static signature scheme exhibited vulnerability to replay attacks.

GPKI-based systems provide strong security guarantees but restrict users to fixed locations due to their reliance on manual verification processes, thereby significantly constraining DePIN’s inherent flexibility. Conversely, more flexible authentication approaches compromise security by simplifying identity confirmation procedures.

### Motivation

Related work demonstrates that blockchain enables lightweight and efficient authentication, whereas GPKI offers robust identity traceability. To concurrently harness the strengths of both technologies and reconcile the trade-offs among lightweight authentication, privacy protection, and identity traceability, this paper introduces government-issued eID cards as a foundational component. By utilizing eID cards to bridge blockchain and GPKI systems, this paper constructs a traceable authentication system for DePIN.

## Preliminaries

### Decentralized physical infrastructure networks

DePIN represents a novel networking paradigm that integrates blockchain technology with IoT devices to construct and manage distributed physical infrastructure^23^. DePIN is a distributed network of IoT devices that are interconnected through a decentralized network of gateways. Each gateway is a physical device that connects to the network and provides a secure communication channel between the devices. The gateways are responsible for routing data between the devices and ensuring data integrity and security. The network is designed to be resilient to failures and vulnerabilities, making it highly reliable and secure.

### Smart contract

The concept of smart contracts was first introduced by Nick Szabo in 1994 to facilitate digital markets^24^. A smart contract is a computer program that automatically executes contract terms. It is designed to carry out, control, or document legally relevant events or actions based on the stipulations of a contract or agreement. This automation reduces the need for intermediaries, streamlines processes, and minimizes the risks of errors or fraud. With the advancement of blockchain technology, smart contracts have been deployed on blockchains.

### Government public key infrastructure

The government public key infrastructure is a framework designed to secure digital communications and identity verification by using cryptographic techniques^25^. It is widely used by governments around the world to provide secure, trusted, and verifiable digital services. GPKI issues digital certificates to users or devices, ensuring the authenticity of identities through public and private key pairs.

A key component of GPKI in many countries is the eID card, a physical smart card with a secure chip to store a user’s private key and digital certificates^26^. The private key is protected by the chip’s hardware security features and cannot be extracted from the card. Users can interact with the card through contact or contactless card readers, which use near field communication technology. After authorizing with PIN, users only need to provide the required data, and the card will automatically generate the digital signature.

The current mainstream signature algorithm for eID cards is RivestShamirAdleman algorithm, such as in Estonia, Finland, Sweden, and Japan^27–29^. However, due to the Return of Coppersmith’s Attack vulnerability in the RSA algorithm^30^, many countries have used or plan to use the Elliptic Curve Digital Signature Algorithm (ECDSA)^28,29^.

### Rivest–Shamir–Adleman (RSA) algorithm

RSA is a widely used public key cryptosystem based on the difficulty of integer factorization^31^. In this system, RSA is employed to generate and verify digital signatures. A typical RSA signature scheme consists of three steps:

- **KeyGen**(*p*, *q*): Generates the public key (*n*, *e*) and private key *d* using two large primes.
- **Sign**(*m*, *n*, *d*): Uses the private key *d* to sign a message *m*, producing signature $\sigma$.
- **Verify**$(\sigma ,n,e)$: Validates the signature $\sigma$ using the public key (*n*, *e*). Outputs *accept* or $\bot$.

This mechanism ensures that only the holder of the private key can produce valid signatures, thus enabling secure authentication.

## System model and security considerations

### System structure

The system architecture comprises four components: government, blockchain, users, and physical infrastructure devices.

*Government*: The government is a trusted Certificate Authority (CA) responsible for the lifecycle of eID, including users’ in-person identity verification, key pair generation, certificate issuance, authentication, and certificate revocation. Only the government can deploy and set up smart contracts. After completing users’ in-person identity verification, the government issues an eID card to users and establishes mappings between the eID public key and the user’s identity in the government database.

*Blockchain*: The blockchain network is operated by trusted government institutions as nodes. The blockchain is responsible for operating two smart contracts, the authentication contract and the certificate revocation list (CRL) contract. The authentication contract stores the government’s public keys for signature verification, enabling automatic authentication and recording results in transaction logs. The CRL contract utilizes hash table structures to store revoked certificates. Only authorized users and nodes can access, read, or write data on the blockchain.

*Users*: Users keep their own eID cards and are required to promptly report to the government if eID cards are lost or stolen. Only the user knows the PIN associated with the eID card, and digital signatures can be generated only after the correct PIN is entered. Users can utilize any blockchain account to submit the required authentication information, which will be verified by the smart contract. Communication between users and physical infrastructure devices is encrypted using the RSA algorithm to ensure secure transmission.

*Physical infrastructure devices*: Each infrastructure device is bound to its owner’s blockchain account and can independently query and get authentication results on the blockchain. Despite performance limitations, the devices support random number generation and RSA encryption.

### System workflow

**Fig. 1:**
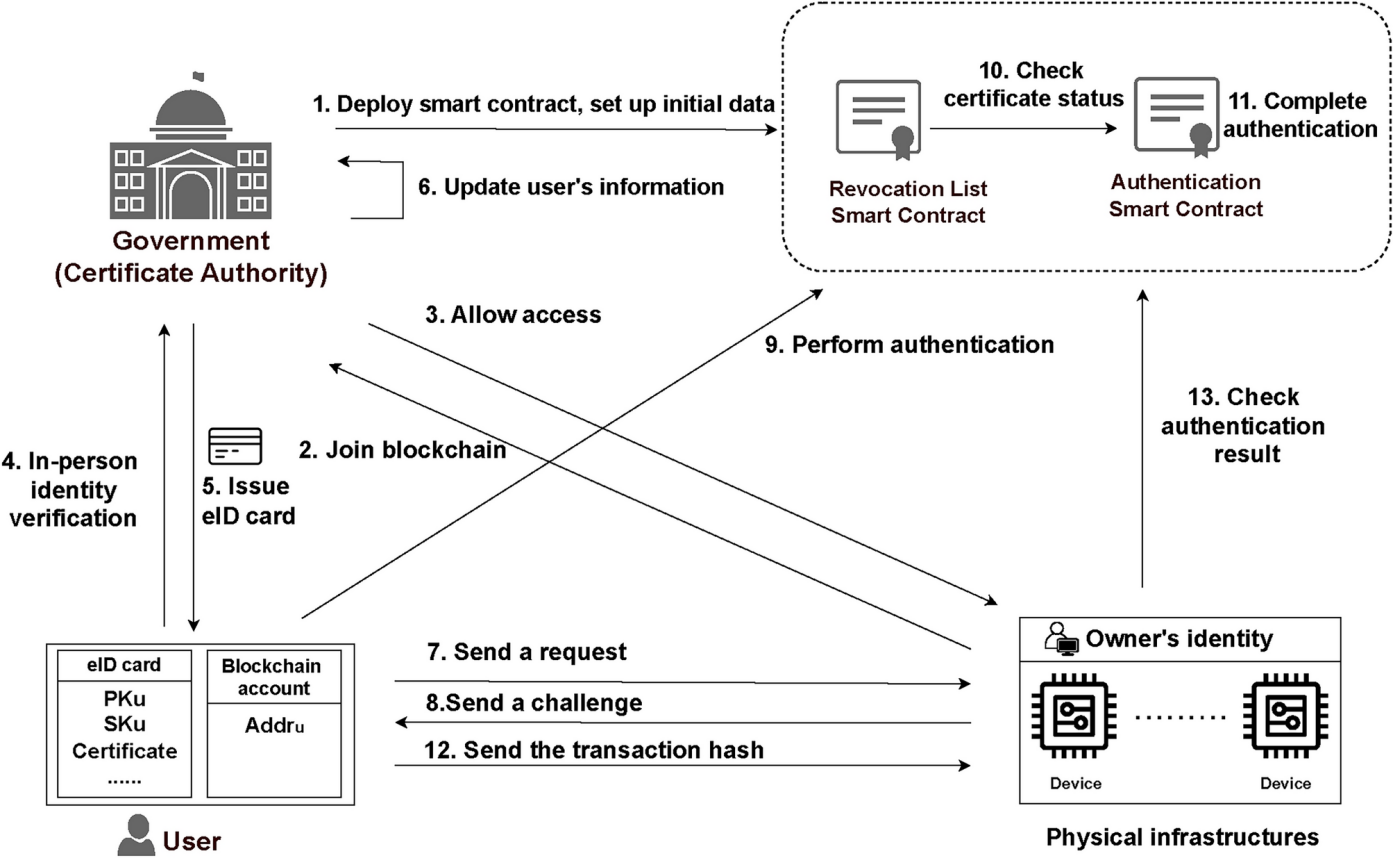
System architecture (This figure was originally created by the authors using draw.io.).

The following steps outline the system process, as shown in the Fig. 1.

1. The government generates a key pair and deploys two smart contracts on the blockchain: authentication and CRL contracts. Set the public key in the authentication contract.
2. The physical infrastructure owner applies to join the blockchain network to access blockchain content.
3. The government authorizes blockchain account access upon verifying the owner’s identity.
4. Users must conduct in-person identity verification at government offices.
5. After confirming the user’s identity, the government generates a key pair and signs the user’s public key with its private key to create a digital certificate. Subsequently, the key pair and certificate are stored in the IC chip of the eID card.
6. The government establishes a mapping relationship between the user’s real-world identity and the card’s public key.
7. The user initiates an access request to a physical infrastructure device and provides the user’s blockchain account address.
8. The physical infrastructure device returns a random challenge number to the user.
9. The user generates a signature using the eID card and initiates an authentication transaction containing the signature and certificate.
10. The smart contract first verifies the certificate status.
11. After confirming that the certificate has not been revoked, proceed to verify both the signature and the certificate. The user’s address, the verification result, and the random number will be recorded in the transaction log.
12. The user sends the transaction hash to the physical infrastructure device.
13. The physical infrastructure checks the verification result from the transaction log to determine access authorization.

### Threat model

The attacker’s purposes are unauthorized access to physical infrastructure devices and determining which user uses the PI device.

The adversary’s abilities are as follows:

1. A Probabilistic Polynomial-Time (PPT) adversary can eavesdrop, intercept, modify, and erase the message communicated between the user, blockchain, and physical infrastructure devices across the public channel. However, the adversary is computationally bounded and cannot break standard cryptographic assumptions such as RSA.
2. The government is regarded as a completely honest entity. Therefore, an adversary cannot obtain data held by the government. However, an adversary can obtain the government’s publicly available public key from a smart contract.
3. Blockchain nodes are viewed as honest but curious entities. Adversaries can access blockchain data to plan malicious behavior.
4. Users are considered honest entities that follow the protocol to authenticate and access PI devices. However, an adversary may act as a user by obtaining an eID card, accessing the blockchain, or compromising the private key of a user’s blockchain account.
5. PI devices are viewed as honest entities that follow authentication protocols strictly. An adversary cannot access PI devices unless the device actively allows it to access.

### Security requirements

The proposed system needs to achieve the following security objectives:

*Identity Verifiability*: The system must ensure the authenticity of all authentication requests. Even when a user’s blockchain account is compromised, attackers cannot access physical infrastructure through identity forgery.

*Privacy Protection*: The system must ensure that data stored and transmitted on the blockchain does not leak user privacy. Even if attackers can eavesdrop on communications or analyze blockchain data, they cannot infer the user using the infrastructure device.

*Non-repudiation*: The system ensures that once a user completes authentication and is granted access to a PI device, they cannot repudiate having initiated the authentication request or used the device.

## Proposed architecture

**Fig. 2:**
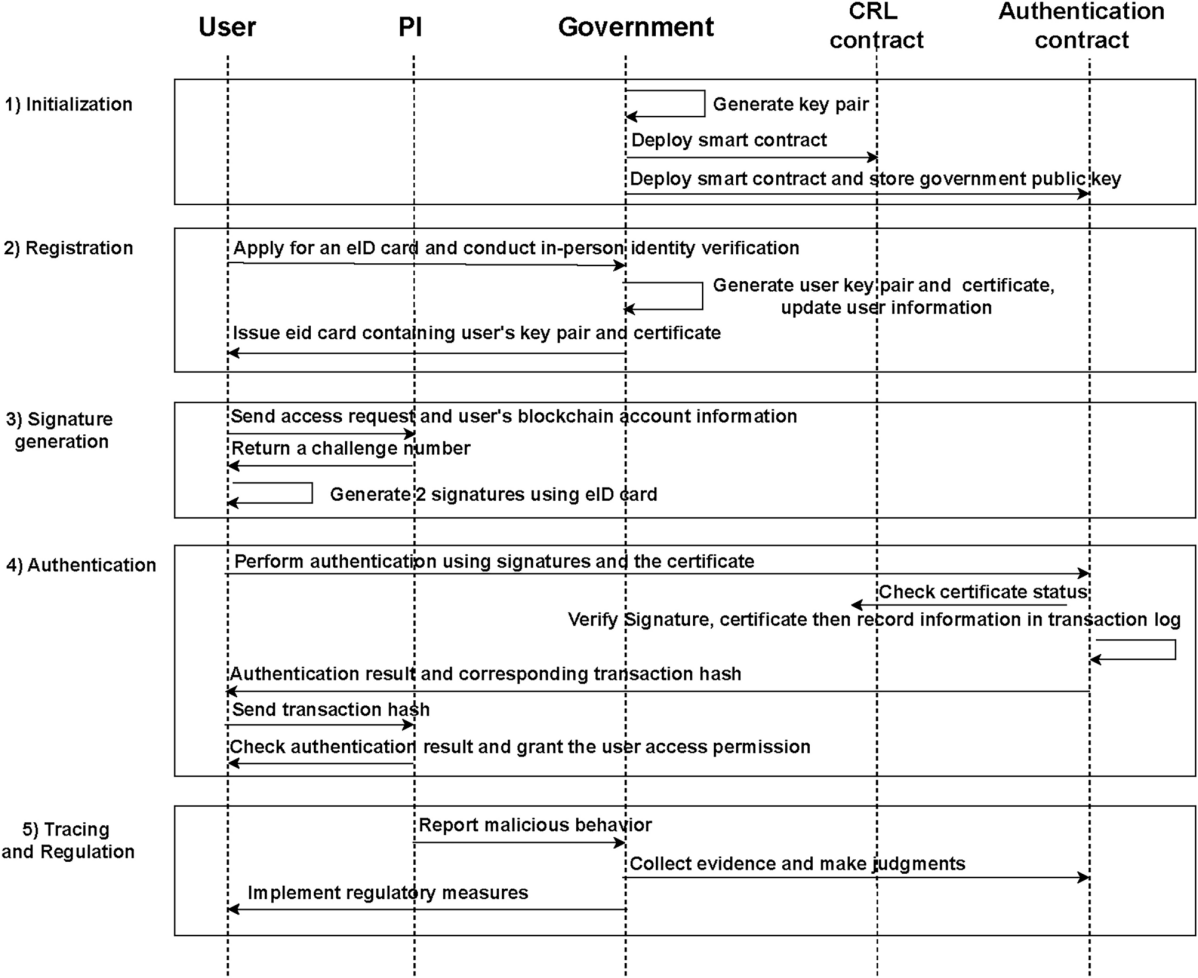
Workflow of proposed system.

The proposed architecture consists of 5 steps, as shown in Fig. 2. Notations are described in Table 1.

**Table 1:** Description of notations used in the system.

Notation	Description
$PK_{ca}$	Government Public Key
$SK_{ca}$	Government Secret Key
$PK_{u}$	Public Key in User’s eID card
$SK_{u}$	Secret Key in User’s eID card
*Cert*	User’s certificate
*isRevoked*	Certificate status
$N_c$	Nonce of challenge
$sign_{ca}(\cdot )$	Signing by government secret Key
$sign_{u}(\cdot )$	Signing by secret Key in eID card
$sig_r$	Signature for response
$sig_a$	Signature for authentication
$Verify(\cdot )$	Signature verifying
$Addr_u$	User’s blockchain account address
*msg*.*sender*	Address who use smart contracts
$SC_{auth}$	Authentication smart contract
$Rid_u$	User’s real-world identity
*HT*	Hash table

### Initialization

The government first generates a key pair, then deploys the CRL and authentication contracts. The government’s public key is stored in the authentication contract and can be viewed by blockchain users.

The CRL contract uses hash tables $HT_c$ to manage revoked certificates. It has three functions that add, query, and update revoked certificates’ status, as shown by the Algorithm 1. At the same time, it sets up a contract address that can query the status of certificates, and can use interfaces to obtain results.

**Algorithm 1:**
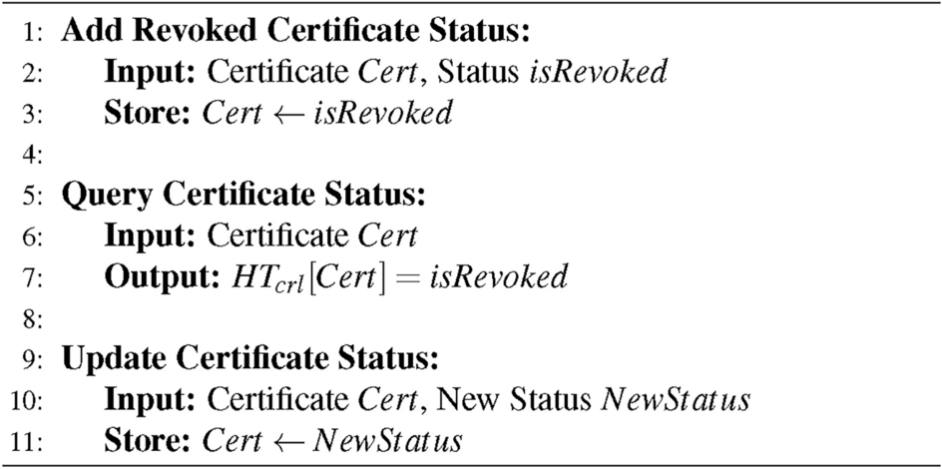
CRL Contract Management

There are two types of certificate status. If a certificate is revoked, the query function will return *True*.

$$\begin{aligned} isRevoked \in \{True, False\} \end{aligned}$$

### Registration

Users must complete registration at government institutions before accessing the system. After real-world identity verification, the system generates each user’s key pair and certificate. The certificate is the government’s signature of the user’s public key. The government controls the validity of user identities through certificate status.

$$\begin{aligned} Cert = Sign_{ca}(PK_u) \end{aligned}$$

Afterwards, the government uses a hash table $HT_{id}$ to manage users’ real-world identities and public keys. Adding and querying functions are shown in the Algorithm 2.

**Algorithm 2:**
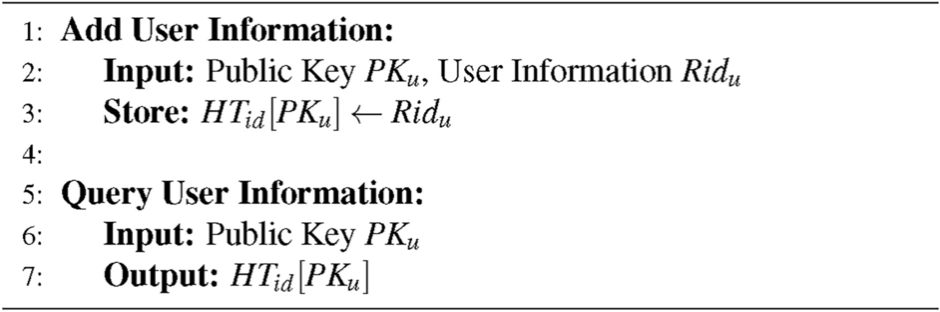
Real-world Identity Management

### Signature generation

To complete authentication, users must generate two types of signatures: a response signature $Sig_r$ and an authentication signature $Sig_a$.

After sending a request to the physical infrastructure device, the user will receive a random number as a challenge. Users must sign in with the challenge number using their eID card to respond to this request.

On the blockchain, each user initiates transactions through a blockchain account. Users generate an authentication signature using an eID card to prove that the blockchain account is being used by themselves.

$$\begin{aligned} Sig_r&= Sign_u({N_c})\\ Sig_a&= Sign_u({PK_u \parallel Addr_u}) \end{aligned}$$

### Authentication

The authentication method relies on signature verification, the process of function *Verify*() is shown as Algorithm 3.

**Algorithm 3:**
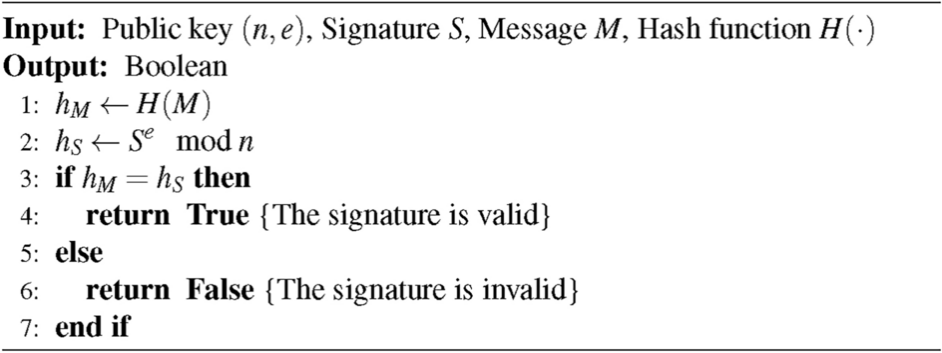
Signature Verification

In smart contracts, *msg*.*sender* represents the address of the transaction sender. Users initiate a transaction through blockchain accounts and provide the necessary information to invoke the smart contract for authentication.

$$\begin{aligned} SC_{auth}(PK_u, Cert, Sig_r, Sig_a, N_c) \end{aligned}$$

The smart contract then executes a series of verification steps to get results: First, it queries the certificate status to confirm it has not been revoked; Second, it verifies the certificate’s authenticity; Third, it verifies the identity verification signature; Finally, it verifies the response signature.

$$\begin{aligned} r_1&= HT_{crl}[Cert]\\ r_2&= Verify(PK_{ca}, Cert)\\ r_3&= Verify(PK_u, PK_u\parallel msg.sender, Sig_a)\\ r_4&= Verify(PK_u, N_c, Sig_r) \end{aligned}$$

Upon completing all verification steps, the smart contract will trigger an event to record the result and information in the transaction log.

$$\begin{aligned} !r_1 \wedge r_2 \wedge r_3 \wedge r_4 = True\\ \downarrow \qquad \qquad \\ Log = \{Addr_u, Success, N_c\} \end{aligned}$$

The physical infrastructure devices retrieve log information based on the transaction hash via a blockchain explorer or interface. The user is granted access upon confirming the blockchain account, authentication results, and challenge number.

### Tracing and regulation

When the owners of physical infrastructure detect malicious behavior that violates service agreements, such as resource abuse, device overuse, or malware installation, the owners can provide evidence to the government, including access logs and records of abuse for adjudication. As a blockchain administrator, the government can utilize smart contract timestamps, authentication results, and other relevant evidence to make a judgment. Upon confirming malicious behavior, the government may use the public key of the eID card to identify the corresponding real-world identity and implement appropriate regulatory measures, such as certificate revocation or legal prosecution.

### Summary

The authentication process relies on signature verification to ensure the authenticity of the user’s identity and prevent impersonation. Identity verification is successful only when all five key elements are correctly validated. The specific elements and corresponding verification materials are as follows.

- User’s real-world identity: *PK* *ca*, eID card
- User’s identity validity: *Cert*, *isRevoked*
- User’s blockchain account: $Sig_{a}$
- Blockchain account’s identity: *msg*.*sender*, $Addr_u$
- Unique access request: $Sig_{r}$

## Security analysis

This section analyzes the correctness and security of the proposed system.

### Correctness

This subsection proves the correctness of authentication in the proposed system.

#### Theorem 1

(Correctness of Authentication) *If a user’s public key is correctly certified by the CA, the certificate is not revoked, and signatures submitted to the blockchain are generated following the exact algorithm provided in section* “Signature generation”. *Additionally, the smart contract is correctly deployed by the CA. Then the smart contract will always successfully authenticate this user*.

#### Proof

Assume, for contradiction, that even though all preconditions are satisfied, the smart contract fails to return True during authentication.

The smart contract performs four verification steps:

Certificate Status Verification:

$$\begin{aligned} r_1&= {HT}_{crl}[{Cert}] = {False} \end{aligned}$$

Since the certificate is not revoked, $r_1$ must pass.

Certificate Verification:

$$\begin{aligned} r_2&= {Verify}(PK_{ca}, {Cert}) = {True} \end{aligned}$$

Because the certificate is correctly signed by the CA and the contract stores the correct $PK_{ca}$, this verification is also correct.

Authentication Signature Verification:

$$\begin{aligned} r_3&= {Verify}(PK_u, PK_u\parallel msg.sender, Sig_a)={True} \end{aligned}$$

When a user uses a blockchain account for authentication, *msg*.*sender* matches the blockchain account address.

$$\begin{aligned} Addr_u = msg.sender \end{aligned}$$

When the user initiates the transaction, the verification result is true.

$$\begin{aligned}&Verify(PK_u, PK_u\parallel msg.sender, Sig_a) \\ =&Verify(PK_u, PK_u\parallel Addr_u, Sign_a)\\ =&Verify(PK_u, PK_u\parallel Addr_u, Sign_u({PK_u \parallel Addr_u})) \end{aligned}$$

The user signed $(PK_u \Vert {Add_u})$ using $SK_u$ as defined in section “Signature generation”, so this verification is correct.

Response Signature Verification:

$$\begin{aligned} r_4&= {Verify}(PK_u, N_c, {Sig}_r) = {True} \end{aligned}$$

The $N_c$ was signed with the correct private key and message. Again, this check should pass.

The authentication succeeds only if:

$$\begin{aligned} \lnot r_1 \wedge r_2 \wedge r_3 \wedge r_4 = {True} \end{aligned}$$

Under the assumption, all conditions are met. Therefore, the smart contract must return *True*. This contradicts the assumption that the contract fails to return True.

Hence, the smart contract will always successfully authenticate the user under the stated conditions. $\square$

### Security

This subsection demonstrates the system’s capability of identity verifiability, privacy protection, and non-repudiation by security analysis.

#### Theorem 2

(Strong authentication) *For a PPT adversary* $\mathcal {A}$, *assume that* $\mathcal {A}$ *takes full control of the user’s blockchain account. The probability that* $\mathcal {A}$ *forges valid signatures* $(Sig_r, Sig_a)$ *and passes smart contract authentication, without the knowledge of the corresponding eID card secret key* $sk_u$, *is negligible*.

#### Proof

Strong authentication implies that even if a user’s blockchain account is stolen, an attacker cannot exploit the victim’s account to access new PI devices, which may lead to untraceable illegal behavior. This paper demonstrates the proof idea as follows: Assume that $\mathcal {A}$ is a PPT adversary that can successfully forge valid signatures $(Sig_r, Sig_a)$ under the proposed authentication scheme without access to the corresponding user’s eID secret key.

This implies that $\mathcal {A}$ is capable of breaking the unforgeability of the RSA signature scheme by generating a valid signature. Hence, the security of strong authentication is reduced to the security of the RSA signature. RSA signature has been proven to be EU-CMA secure against forgery^32^. Therefore, for the adversary $\mathcal {A}$, the probability of being authenticated by the smart contract should be negligible. $\square$

#### Theorem 3

(User privacy) *A PPT adversary* $\mathcal {A}$ *cannot learn the real-world identity of a user by eavesdropping the communication between an honest user and a PI device, and interacting with the blockchain*.

#### Proof

The entry point of attacking user privacy in the proposed system lies in the relationship between the blockchain address $Addr_u$ and its owner. Although blockchain is usually claimed to be anonymous, most blockchain architectures do not provide a solid method to prevent one’s blockchain address from being known. As a consequence, a user’s address can inevitably be linked to their real-world identity. For example, transferring crypto money to a real-world friend easily exposes addresses to each other.

In the proposed scheme, the user privacy requires that even if a user’s real-world identity and blockchain address are known by the public, an attacker cannot know which PI device is occupied by this user. Note that a user sends two distinct messages to the PI device in the proposed system, which contain $Addr_u$ and a blockchain transaction hash that also reveals $Addr_u$. Intuitively, the privacy is broken if either of the two messages can be learned. To this end, assume that $\mathcal {A}$ is a PPT adversary that can learn the plaintext of any of the two encrypted messages, without the knowledge of the PI device’s secret key.

This implies that $\mathcal {A}$ is able to break the confidentiality of the encrypted message and recover the plaintext from the ciphertext without any knowledge of the secret key. It is easy to see that $\mathcal {A}$ can be employed to break the semantic security of RSA encryption. Thus, breaking the user privacy of the proposed system is reduced to breaking the semantic security of RSA encryption. However, RSA encryption is proven to be semantic secure^33^. Therefore, the adversary $\mathcal {A}$ cannot learn the user corresponding to a PI device with a non-negligible probability. $\square$

#### Theorem 4

*If smart contracts are correctly deployed, a PPT adversary* $\mathcal {A}$* cannot be authorized to access a PI device by attacking the blockchain*.

#### Proof

An adversary may attempt to gain access from the blockchain layer by modifying blockchain data: Blockchain data is maintained by government-authorized nodes using a multi-node consensus mechanism. Although theoretically, an adversary could manipulate the data by controlling the majority of blockchain nodes. This is practically infeasible, as all nodes are operated by trusted government and institutional entities. Therefore, $\mathcal {A}$ cannot be authorized to access the PI device by altering the smart contract or authentication logs. $\square$

#### Theorem 5

(Non-repudiation of Access)* If a user successfully completes authentication and gains access to a PI device, the user cannot repudiate it after performing this action*.

#### Proof

When a user completes authentication, the smart contract stores the authentication result on the blockchain.

According to Theorem 2, only the user possessing the eID card can generate the response signature $Sig_r$.

According to Theorem 4, the blockchain data cannot be modified. Therefore, the user’s real-world identity is consistently a blockchain account identity. The user cannot repudiate their interaction with the system or the access action taken on the device, thereby ensuring accountability and non-repudiation. $\square$

## Performance evaluation

This paper focuses on the authentication requirements in DePIN and implements three experiments to comprehensively evaluate the system from the perspectives of hardware, batch verification, and cost.

### Hardware performance

**Fig. 3:**
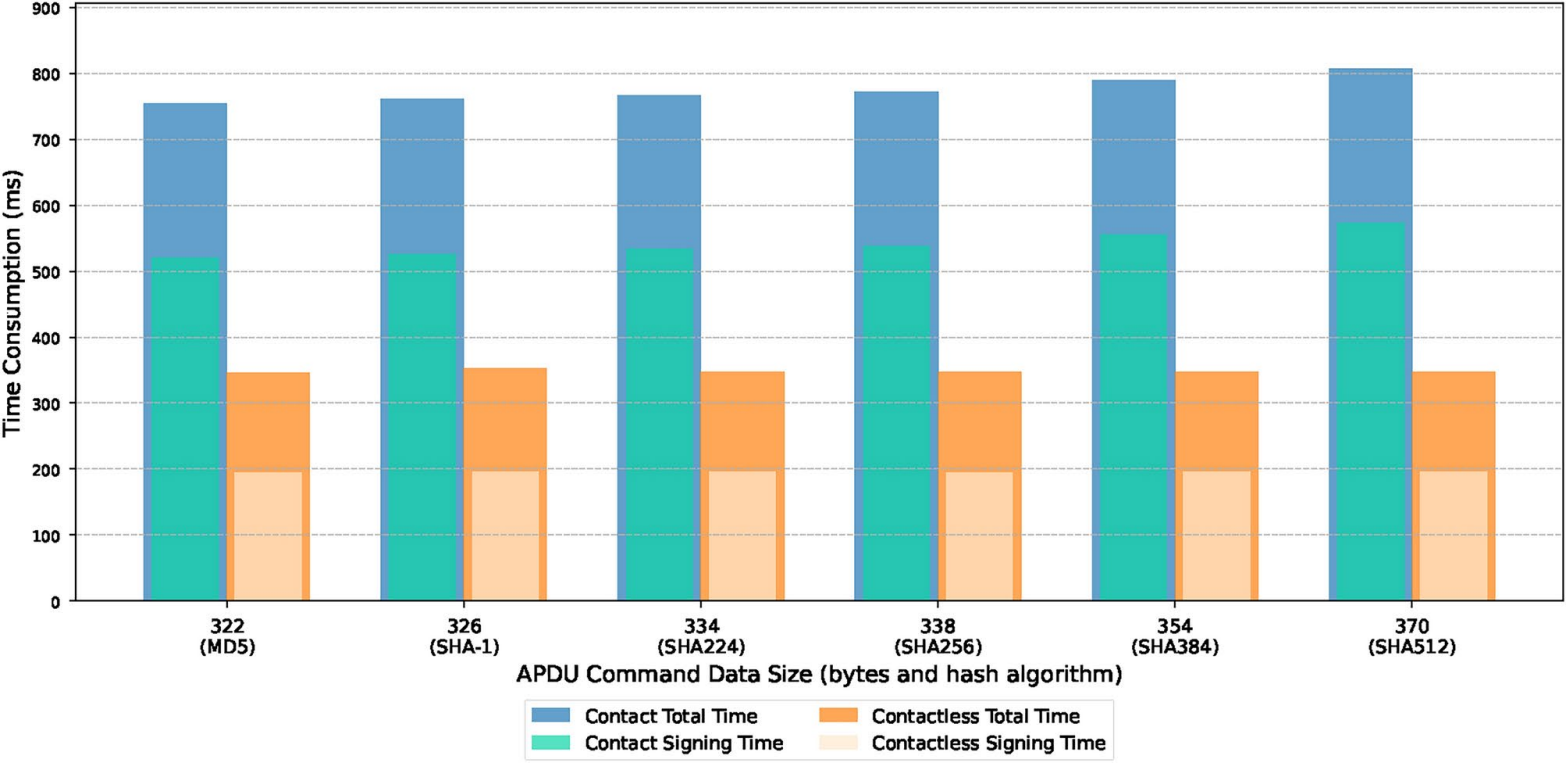
Performance evaluation of signature generation using Japanese government-issued eID cards using various hash algorithms.

Experiments were conducted using a Japanese government-issued electronic ID card (J-eID) with the RSA-2048 algorithm. The card stores the public key, private key, and cryptographic module, and implements access control through PIN verification. The J-eID card’s built-in cryptographic module provides digital signature functionality following the Public Key Cryptography Standards (PKCS) #7 standard, with communication handled through the PKCS #11 interface. The user generates a signature using the card’s encryption module through the application protocol data unit (APDU) command via the card reader. The signature process includes PIN verification and signature generation, and each step requires communication with the eID card using APDU instructions. The total time and signature time under different message digest algorithms were tested, as shown in the Fig. 3.

The results indicate that the total time increases with data size. Under the same data conditions, contact card readers consume more time than contactless card readers. This indicates that the card reader’s performance significantly impacts the time required for signature generation.

**Fig. 4:**
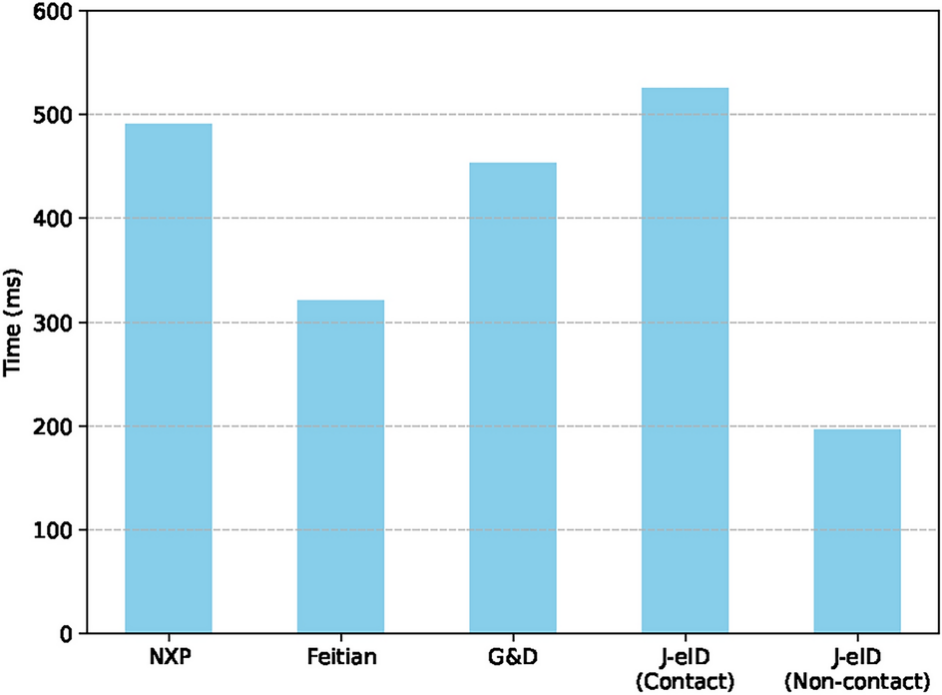
Comparative analysis of SHA-1 signature generation performance among different types of eID smart cards.

The signature time of J-eID was compared and analyzed with the work of Rezaeyaleh et al.^34^, as shown in Fig. 4. Although the SHA-1 algorithm is now considered insecure for encryption, the comparison results provide valuable reference for the overall performance characteristics of different smart card implementations. The results indicate that the performance of J-eID card in using contact card reader is comparable to NXP card, although its performance is relatively lower compared to Feitian and G&D cards. It is worth noting that the J-eID card exhibits excellent performance in the contactless card reader compared to similar products, with significantly reduced processing time.

### Batch verification performance

To evaluate the authentication performance of the system in high concurrency scenarios, throughput tests were conducted in a single node blockchain environment under different numbers of requests. The node is configured with Intel Core i5-9600KF CPU and 32GB RAM, generating transaction requests using the Hardhat framework and running them in the Anvil testing environment. RSA signatures were generated by a J-eID card.

To further evaluate the compatibility of this system with eID cards from other countries, the analysis is extended to include the ECDSA algorithm alongside RSA. This decision is informed by previous research^15^, which employs ECDSA in a system analogous to ours. The generation of ECDSA signatures is simulated using MetaMask and Web3 interfaces. The experiment tested the performance of two verification methods, RSA and ECDSA, and the certificate state storage function.

**Fig. 5:**
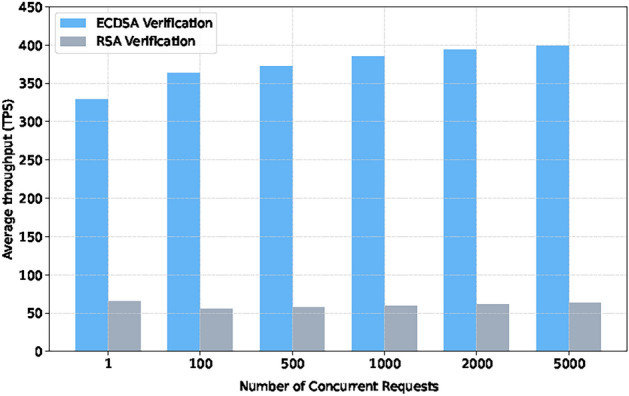
Throughput comparison between RSA and ECDSA signature verification under different concurrent transaction.

**Fig. 6:**
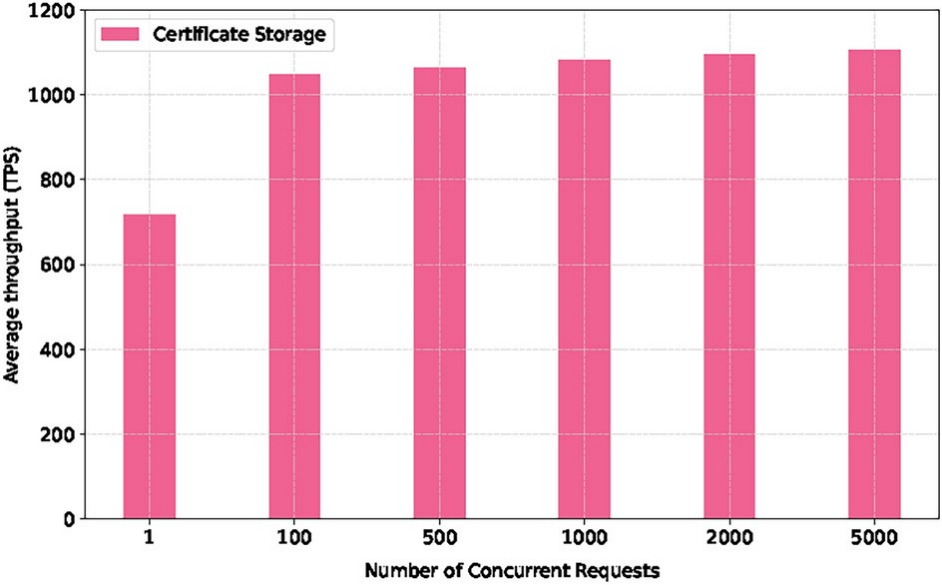
Certificate storage throughput of local blockchain.

As illustrated in Figs. 5 and 6, the experimental results show that the throughput of both verification methods increases with the increase of request quantity. Still, the performance of ECDSA is significantly better than RSA. Under the maximum concurrent requests, ECDSA’s throughput reaches 400 TPS, while RSA remains at around 55 TPS. In addition, the throughput of the certificate status storage function reaches 1080 TPS, which can meet the real-time update requirements of certificate status for large-scale devices.

The main reason for the performance difference is the difference in signature length. The RSA signature has a length of 2048 bits, which requires greater storage and transmission overhead; The ECDSA signature is relatively short, only 512 bits, making its verification process more efficient. This result indicates that ECDSA has significant scalability advantages in high concurrency verification scenarios.

**Fig. 7:**
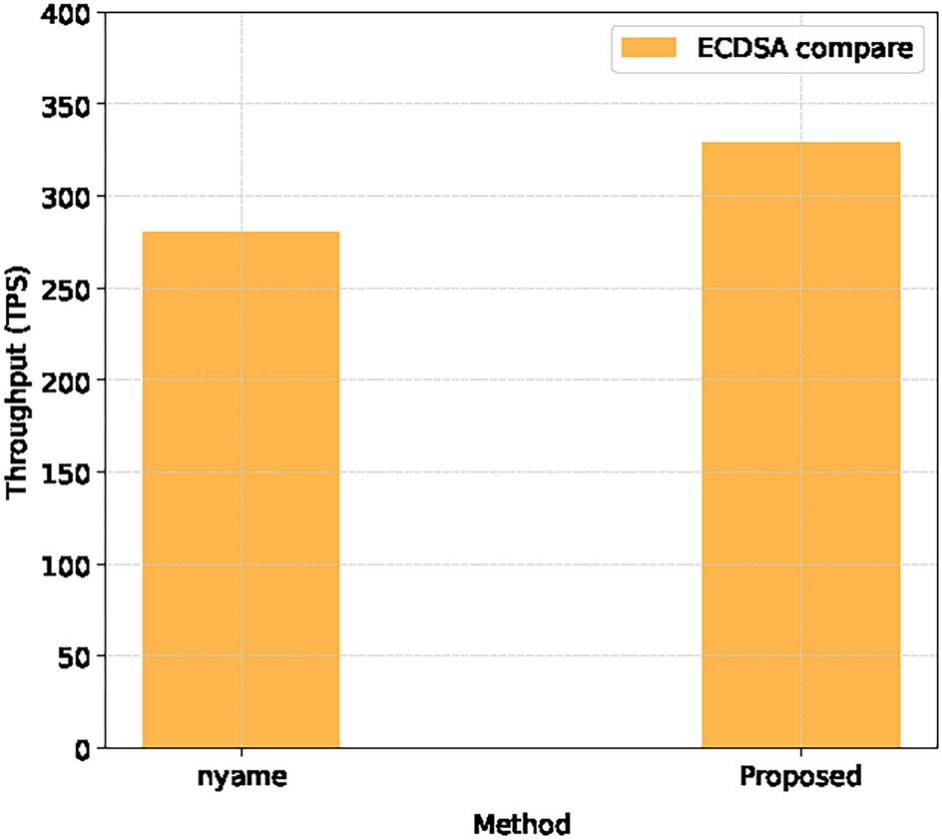
Throughput comparison of local blockchain.

Compared with Nyame et al.’s system^35^, this system achieved higher throughput under the ECDSA algorithm, as shown in the Fig. 7.

### Cost performance

To evaluate the performance of the system in terms of resource efficiency, the gas consumption performance of RSA and ECDSA algorithms was tested in the Anvil local blockchain network and Sepolia test network, as shown in Table 2. The experiment covers multiple operations of smart contracts, including contract deployment, certificate management, and identity verification.

**Table 2:** Comparison of gas costs in different environments.

	Anvil	Sepolia	Nyame et al.^35^	Garba et al.^18^
Revocation List Contract Deployment	179,259	217,594	–	13,600,000
Revoked Certificate Storage Operation	44,690	45,738	–	68,000
RSA Authentication Contract Deployment	925,968	1,352,826	–	–
RSA Public Key Registration	249,443	250,562	–	–
RSA Authentication Operation	174,259	348,519	–	–
ECDSA Authentication Contract Deployment	844,139	1,202,510	1,560,740	–
ECDSA Public Key Registration	44,112	44,032	–	–
ECDSA Authentication Operation	57,271	58,120	97,213	–

The experimental results show that the ECDSA validation method exhibits higher gas efficiency in all operations. For example, in authentication operations, ECDSA reduces gas consumption by 67% compared to RSA, and reduces it by 82% in critical management operations. The gas consumption for contract deployment has also significantly decreased, with ECDSA reducing by about 11% compared to RSA. In addition, compared with the study by Nyame et al.^35^, the consumption of ECDSA-based authentication operations in this system has been reduced by 40% on Sepolia.

The difference in gas consumption is not only related to the complexity of the algorithm, but the signature length is also a key factor. The signature length of RSA is 2048 bits, which is longer than ECDSA, resulting in higher resource requirements for storage and computation. Therefore, in scenarios of frequent operations or high throughput, ECDSA exhibits significant economic advantages.

## Discussion

In this section, this system is compared with related work. Table 3 summarizes the feature comparison.

**Table 3:** Feature comparison of authentication systems.

	Mao et al.^10^	Rahat et al.^12^	Paez et al.^22^	Almadhoun et al.^36^	Liu et al.^15^	Proposed
Automated Authentication	✓	✗	✗	✗	✓	✓
Multiple Encryption Algorithms	✗	✗	✗	✓	✗	✓
Compatibility with Performance	✓	✓	✗	✓	✓	✓
Secure Key Storage in Hardware	✓	✗	✓	✗	✓	✓
No User Information on Blockchain	✗	✓	✓	✗	✓	✓
Identity Tracing	✗	✗	✗	✗	✗	✓

Table 3 focuses on the comparative analysis of the proposed solution and the existing system in two key aspects: system functionality and security. By combining government-issued eID cards with blockchain, this system ensures the reliability of signatures, certificates, and smart contracts.

From a functional perspective, the proposed solution leverages smart contracts to automate the processing of signatures and certificates, thereby eliminating the need for manual intervention by government officials or intermediary nodes. The flexibility of this system enables it to support multiple signature algorithms, making it adaptable to various eID standards worldwide. Furthermore, this solution minimizes computational overhead on constrained devices, ensuring applicability across a wide range of DePIN environments. However, the deployment of such a system remains contingent upon the availability of government-issued eID cards, which may restrict its adoption in regions where this infrastructure is underdeveloped. Although the proposed system significantly enhances authentication security and efficiency, it depends on the integrity of the CA and the blockchain network. If the CA is compromised or the blockchain experiences a security breach, attackers could manipulate authentication records.

Regarding security, this solution employs eID cards to store the certificate to reduce blockchain storage requirements. Compared to Mao et al.’s work^10^, eID cards are easy to deploy and can accommodate a large user base while maintaining security. By avoiding the storage of sensitive information on the blockchain, this solution mitigates the privacy risks identified in Mao et al.’s work^10^ and Almadhoun et al.’s work^36^. Furthermore, the method described in Paez et al.’s work^22^ is restricted to tracking user transaction records. This system not only ensures the traceability of malicious behaviors but also enables the government to implement robust regulatory measures.

If the existing systems are applied to DePIN, there are three fundamental trade-offs: (1) the trade-off between the high cost of on-chain storage and the centralization of off-chain databases. (2) the trade-off between user privacy and identity traceability. (3) the trade-off between flexibility of authentication and security. These limitations underscore the need for a traceable authentication approach specifically tailored to the unique characteristics of DePIN. This paper proposes and implements a traceable authentication system that leverages government-issued eID cards to enable secure identity verification and effective governance of malicious behaviors.

## Conclusion

To address the security issue of malicious behavior in DePIN, this paper proposes a traceable authentication system that integrates blockchain technology with GPKI. The system leverages the IC chip of the eID card to generate signatures and securely store user keys, thereby significantly reducing the storage overhead associated with blockchain. Smart contracts are employed to verify certificates and user signatures, while the public key is recorded in the transaction log to ensure traceability of user identity. Security analysis confirms that the system satisfies essential security properties, including identity verifiability, privacy protection, and non-repudiation. The proposed system is resilient to common attacks such as replay attacks, signature forgery, and data tampering. Performance evaluations show that the system achieves high throughput and reduces authentication costs by up to 40% compared to existing solutions. By reconciling the trade-offs between security, scalability, and privacy, the proposed framework offers a practical and robust solution for identity authentication in DePIN environments. It further lays a foundation for applications in smart cities, digital public infrastructure, and secure IoT access control.

## Data Availability

The data used in this study can be obtained from the corresponding author upon reasonable request.
